# Myco-Suppression Analysis of Soybean (*Glycine max*) Damping-Off Caused by *Pythium aphanidermatum*

**DOI:** 10.3390/plants10040788

**Published:** 2021-04-16

**Authors:** Shaban R. M. Sayed, Shaimaa A. M. Abdelmohsen, Hani M. A. Abdelzaher, Mohammed A. Elnaghy, Ashraf A. Mostafa, Fatemah F. Al-Harbi, Ashraf M. M. Abdelbacki

**Affiliations:** 1 Electron Microscope Unit, Collage of Science, King Saud University, Riyadh 11451, Saudi Arabia; shmohamed@ksu.edu.sa; 2 Physics Department, Faculty of Science, Princess Nourah Bint Abdulrahman University, Riyadh 84428, Saudi Arabia; ffalharbi@pnu.edu.sa; 3 Department of Biology, Collage of Science, Jouf University, Sakaka 42421, Saudi Arabia; hmdaher@ju.edu.sa; 4 Department Botany and Microbiology, College of Science, Minia University, El-Minia 61519, Egypt; mohamed.elnag1hi@mu.edu.eg; 5 Botany and Microbiology Department, College of Science, King Saud University, Riyadh 11451, Saudi Arabia; asali@ksu.edu.sa; 6 Plant Pathology Department, Faculty of Agriculture, Cairo University, Giza 12613, Egypt; amaeg@hotmail.com

**Keywords:** biological control, biomaterials, damping-off, *Glycine max*, *P. oligandrum*, *P. aphanidermatum*

## Abstract

The role of *Pythium oligandrum* as a biocontrol agent against *Pythium aphanidermatum* was investigated to avoid the harmful impacts of fungicides. Three isolates of *P. oligandrum* (MS15, MS19, and MS31) were assessed facing the plant pathogenic *P. aphanidermatum* the causal agent of *Glycine max* damping-off. The tested *Pythium* species were recognized according to their cultural and microscopic characterizations. The identification was confirmed through sequencing of rDNA-ITS regions including the 5.8 S rDNA. The biocontrol agent, *P. oligandrum*, isolates decreased the mycelial growth of the pathogenic *P. aphanidermatum* with 71.3%, 67.1%, and 68.7% through mycoparasitism on CMA plates. While the half-strength millipore sterilized filtrates of *P. oligandrum* isolates degrade the pathogenic mycelial linear growth by 34.1%, 32.5%, and 31.7%, and reduce the mycelial dry weight of the pathogenic *P. aphanidermatum* by 40.1%, 37.4%, and 36.8%, respectively. Scanning electron microscopy (SEM) of the most effective antagonistic *P. oligandrum* isolate (MS15) interaction showed coiling, haustorial parts of *P. oligandrum* to *P. aphanidermatum* hyphae. Furthermore, *P. oligandrum* isolates were proven to enhance the germination of *Glycine max* seedling to 93.3% in damping-off infection using agar pots and promote germination of up to 80% during soil pot assay. On the other hand, *P. oligandrum* isolates increase the shoot, root lengths, and the number of lateral roots.

## 1. Introduction

Soybean (*Glycine max* L.) is one of the most common widely cultivated food species in the world [1]. Root rot diseases of *G. max* are widespread in the world and are usually recognized as the main constraint to decrease both yield and quality [2,3]. *Pythium* as a causal agent for *Glycine max* root rots has been mentioned as being the main reason leading to soybean yield losses in numerous countries [4,5]. *Pythium aphanidermatum* has been reported as the species most frequently causing root rot diseases for the greatest variety of crops [4]. The use of chemical fungicides controlling such diseases could lead to degenerating human health, environmental pollution, and the development of pathogen resistance to fungicides. Biological methods are needed for plant protection which are less dependent on chemicals and are more environmentally favorable [5,6].

*Pythium* is a complex genus, spread worldwide including over 200 species with a wide host range and maintaining in a variety of soil and water environments [7,8]. Several researchers have explained that *Pythium oligandrum* efficiency in controlling plant disease, some of them reported that the antagonistic of *P. oligandrum* enhance plant immune systems through secretion of an elicitin-like protein that induces plant resistances against different phytopathogenic fungi, avoiding their infection. The biocontrol activity of *P. oligandrum* attributed to directly attack a group of soil-borne fungal pathogens through; (1) mycoparasitism, (2) colonizing rhizosphere of crop plants and competing for space and nutrients, (3) production of an auxin like tryptophan and indolacetaldhyde, which convert to tryptamine (TNH2) that promote plant growth, and (4) secretion of oligandrin and elicitin-like protein activate plant immune-system that increase crop protection against phytopathogenic microbes [4,9,10,11,12]. A similar mode of action observed by other biocontrol agents like *Trichoderma harzianum* induces systematic resistance in *G. max* plants and enhances plant growth through elicitation of bioactive metabolites [13,14,15].

Horner et al. [16] explained the mycoparasitic ability of *P. oligandrum* through coiling *P. oligandrum* hyphae around phytophthora hyphae and by secretion of glucanases, cellulose, chitinase and, protease enzymes which degraded the pathogen cell wall. *P. oligandrum* used in biocontrol of black scurf in potato as the hyphal of biocontrol agent had colonized the sclerotia and established close contact by coiling around the *R. solani* [17]. *Phytium oligandrum* has taken serious attention as a biocontrol agent against damping-off diseases, especially those caused by *P. aphanidermatum* [8,16,18,19].

No attempts have been made to evaluate *P. oligandrum* as a biocontrol agent against *P. aphanidermatum*, the causal factor of *G. max* seedlings damping-off in Egypt and Saudi Arabia. The present work was, therefore, aimed at evaluating the efficiency of using *P. oligandrum* in the biocontrol of soybean seedling damping-off caused by *P. aphanidermatum*.

## 2. Results

**Figure 1:**
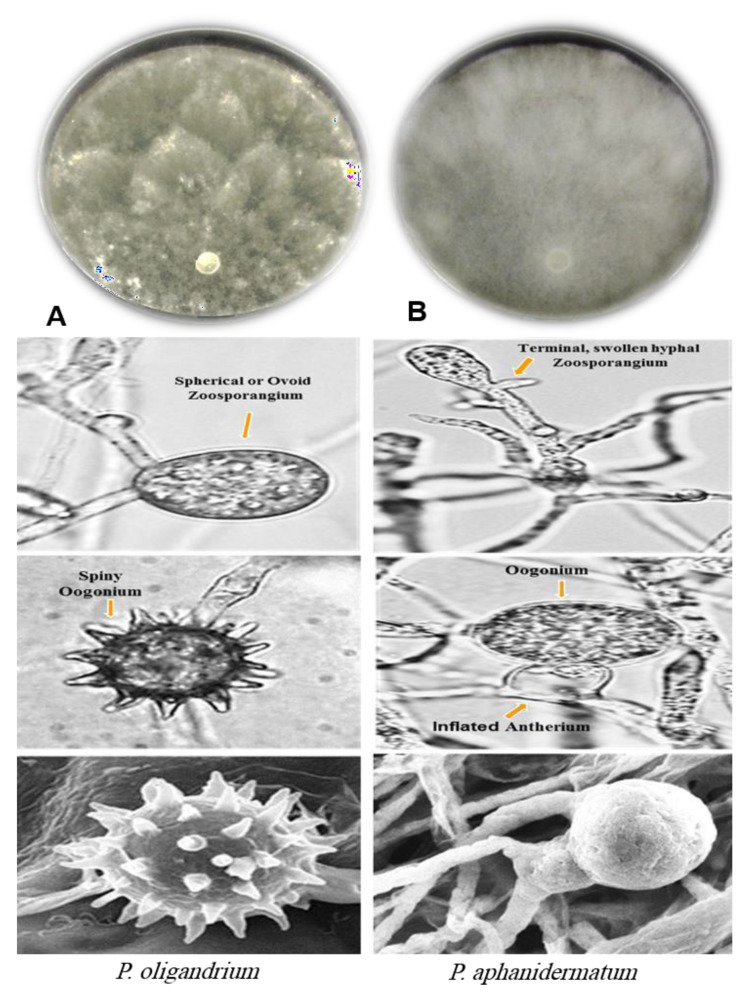
Morphological characterizations of *Pythium* isolates on PDA, light, and scanning electron microscope. (**A**) *P*. *oligandrum* and (**B**) *P*. *aphanidermatum*.

Isolation and identification of biocontrol *Pythium* isolates showed the presence of three strains of *P. oligandrum* (MS15, MS19, and MS31) obtained from the rhizosphere soil of *Raphanus sativus*, *Eruca sativa,* and *Allium cepa*, respectively, during the survey of *Pythium* species in El-Minia Governorate, Egypt. These three isolates were identified morphologically as *P. oligandrum* Drechsler (Figure 1a). In addition, the sequencing of rDNA-ITS for these isolates was closely related to *P. oligandrum* with 99.9% similarity to Genbank accession number (AY986954.1).

Isolation of pathogenic strains from the infected roots and seedlings of *G. max* indicated that *P. aphanidermatum* was the only fungus present in infected tissues. The morphological identification of pathogenic *P. aphanidermatum* was illustrated in Figure 1b and the genetic criteria of this isolate were closely related to *P. aphanidermatum* with 99.9% similarity to Genbank accession number (AB274404).

Antagonistic activity of *P. oligandrum* isolates (MS15, MS19, and MS31) against the pathogenic *P. aphanidermatum* using dual culture technique was reported in Table 1. The bioactivity assay showed that *P. oligandrum* isolates decreased the mycelial growth of *P. aphanidermatum* and the MS15 was the highest one after 3 days of incubation and reduced the growth by 67.2%. Moreover, the reduction in the growth of pathogenic *P. aphanidermatum* was increased by increasing the incubation time, where the inhibition reached 71.3% for MS15 after 6 days of incubation, while the results of the isolates (MS19 and MS31) were less effective in all treatments (Table 1).

The effect of half-strength culture filtrates of bioagent *P. oligandrum* isolates on mycelial growth of pathogenic *P. aphanidermatum* using solid media revealed that the millipore sterilized half-strength filtrates of *P. oligandrum* isolates were more effective in reducing the mycelial growth of *P. aphanidermatum* than that of autoclaved cultured filtrate recording inhibition of mycelial growth of *P. aphanidermatum* by 34.1%, 32.5%, and 31.7%, respectively (Table 2).

The effect of culture filtrates of *P. oligandrum* isolates on the mycelial dry weight of *P. aphanidermatum* using liquid medium reported in Table 3. The millipore sterilized half-strength filtrates of *P. oligandrum* isolates reduce the mycelial dry weight of *P. aphanidermatum* more effectively than autoclaved filtrate recording 40.1%, 37.4%, and 36.8%, respectively, and the height reduction was reported by *P. oligandrum* (MS_15_).

**Figure 2:**
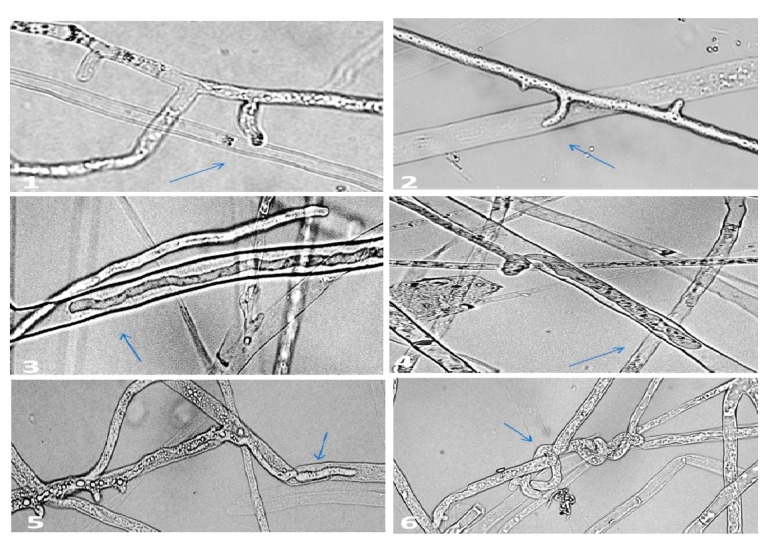
Hyphal interactions between *P*. *oligandrum* (MS15) and *P*. *aphanidermatum* using a light microscope: (**1**) and (**2**) showing infection pegs of *P. oligandrum*; (**3**) and (**4**) showing hyphae of *P*. *oligandrum* grow inside hyphae of *P*. *aphanidermatum*; (**5**) and (**6**) arrows pointed to *P. oligandrum* twisted on hyphae of *P*. *aphanidermatum*.

**Figure 3:**
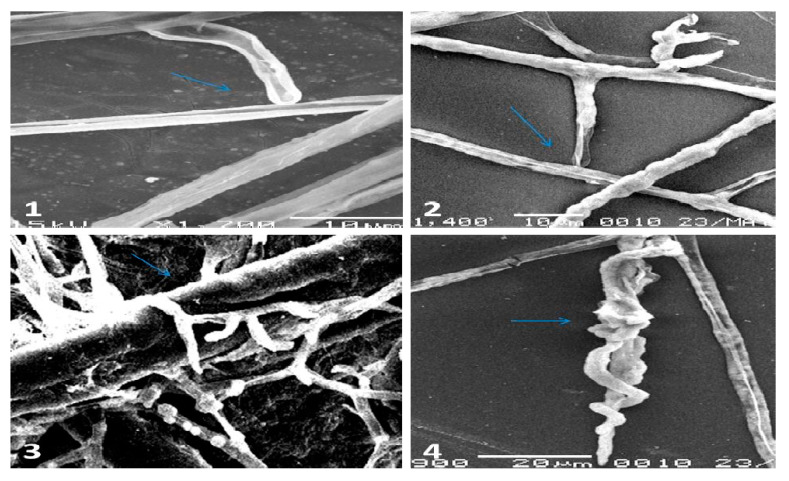
Hyphal interactions between *P. oligandrum* (MS15) and P. aphanidermatum using SEM: (**1**) and (**2**) showing infection pegs of *P. oligandrum*; (**3**) and (**4**) showing torsion of *P. oligandrum* hyphae *P. aphanidermatum* and the arrow pointed to hyphal collapse and turgor loss of *P. aphanidermatum* hyphae.

Interaction between the highly effective isolate of *P. oligandrum* (MS15) and *P. aphanidermatum* using a light microscope and scanning electron microscope (SEM) were provided in Figure 2 and Figure 3. The characterization of coiling, haustorial branches, infection pegs, and penetration of *P. oligandrum* to *P. aphanidermatum* hyphae was shown in the contact area between the two Pythia. *Pythium oligandrum* attacked *P. aphanidermatum* by entangling its hyphae with either thin haustorial arms or infection pegs reaching finally to its total destruction. These characters of mycoparasitism were showed clearly at the early stages of the contact region between the two colonies of *P. oligandrum* and *P. aphanidermatum* on the surface of Corn Meal Agar CMA plates.

**Figure 4:**
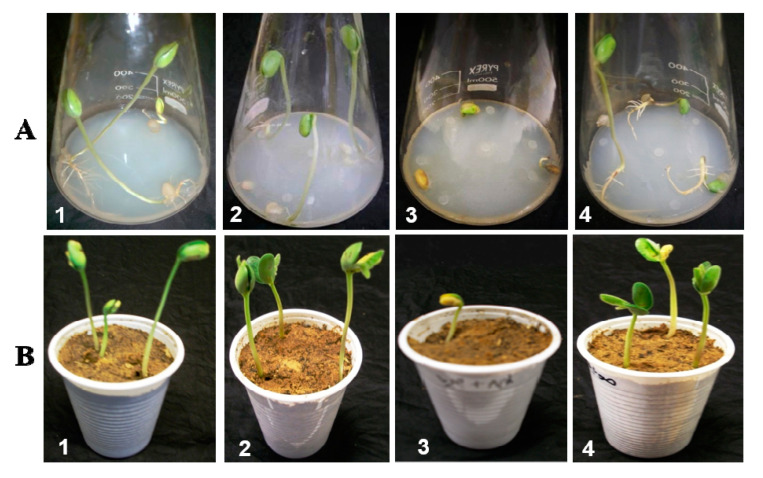
Bioactivity of *P. oligandrum* (MS_15_) on *P. aphanidermatum* through the cultivation of *G. max* seeds on 2% water agar medium (**A**) and soil pots (**B**): 1, seeds cultivated alone as a control; 2, seeds cultivated with *P. oligandrum* alone; 3, seeds cultivated with *P. aphanidermatum* alone; 4, seeds cultivated with both of *P. oligandrum* and *P. aphanidermatum.*

Pathogenicity of *P. aphanidermatum* and its possible control measure by *P. oligandrum* isolates (MS15, MS19, and MS31) in both agar bottles and soil pots assay were illustrated in Table 4 and Figure 4. It was found that the pathogenic isolate of *P. aphanidermatum* was highly pathogenic causing 100% and 86.7% seedling damping-off when the pathogen cultured with seedlings alone in both 2% WA and soil pots respectively. In contrast, the three isolates of the biocontrol *P. oligandrum* isolates were detected to be non-pathogenic to *G. max* seedlings and also improve the seedling growth more than the control treatment. On the other hand, the application of the biocontrol *P. oligandrum* isolates inhibited the seedling damping-off by 93.3%, 87.2%, and 86.7% in 2% WA assay and 80%, 75.1%, and 76.7%, respectively in soil pots compared with the control. Moreover, *P. oligandrum* isolate (MS15) was the most effective isolate in both treatments.

**Figure 5:**
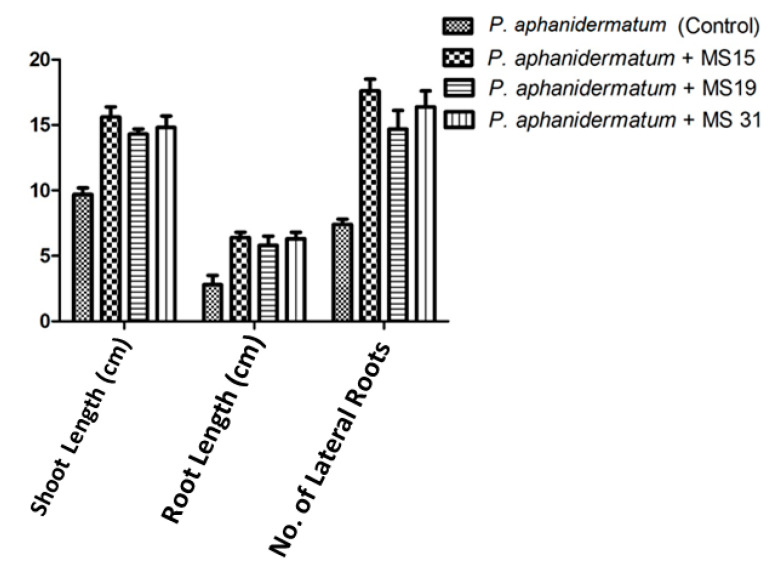
Effects of *G. max* seed treatment with the pathogenic *P. aphanidermatum* and three isolates of *P. oligandrum* (MS15, MS19, and MS31) on growth seed parameters. (Data are means of (3) replicates; values are significantly compared with control at *p* ≤ 0.05).

**Figure 6:**
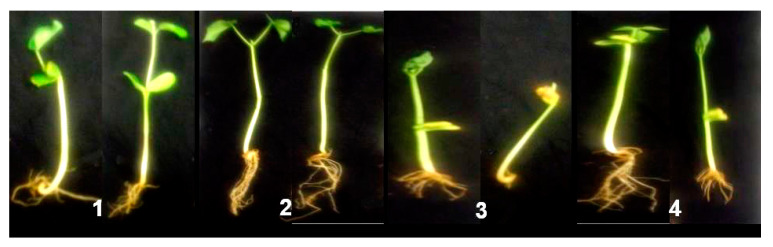
Growth of *G. max* seedling parameters: (**1**), uninfected seedlings (negative control); (**2**), seedlings cultivated with *P. oligandrum* (positive control); (**3**), seedling infested with *P. aphanidermatum* alone and (**4**), seedling infested with both *P. oligandrum* and *P. aphanidermatum*.

The effect of *P. oligandrum* on the growth parameters of the *G. max* seedling was recorded in Table 5 and illustrated in Figure 5 and Figure 6. The results showed that the growth parameters of the treated plants with *P. oligandrum* isolates were enhanced by increasing the shoot and root lengths and increasing the number of lateral roots in all treatments of bioagent isolates. The phytopathogenic *P. aphanidermatum* affected seedling growth of *G. max* seeds and reduced their shoot and root length by 43.3% and 34.9%, respectively, as shown in Figure 6 when compared with the healthy plants (no *Pythium*) in Table 5.

## 3. Discussion

Root damping-off diseases are widely disturbed all over the world and are recognized as a serious problem to soybean cultivators, as they significantly decrease the bean yield. Several control measures of the disease are available, including fungicidal control, enhancement of genetic resistance, which may lead to deteriorating human health [5,20]. In Egypt, there is no active control method to reduce damping-off of many crops and vegetable plants caused by *P. aphanidermatum* although, that pathogenic is widely distributed throughout the country [17,18,21,22]. However, fungicides are regularly used by farmers to manage such diseases without obvious results [6,23]. *Pythium aphanidermatum* was isolated in this study as the causal agent of *G. max* damping-off and the pathogenicity test concerned with the pathogenic isolate of *P. aphanidermatum* was highly virulent to *G. max* seedlings causing 100% damping off. In contrast, the three isolates of *P. oligandrum* (MS15, MS19, and MS31) were found to be non-pathogenic and promote seed germination [24,25]. This result was in agreement with the result of Nzungize et al. [20,26].

Biological control of damping-off diseases is a complicated process as a result of the pathogens occurs surroundings the rhizosphere interface. The rhizosphere is indicated by intense microbial action including, a high population of microorganisms, salt concentrations, and osmotic and water potential [27]. The efficient microorganisms for biological control of phytopathogenic fungi include some *Pythium* spp. as *P. oligandrum.* The biocontrol agent can preserve the plant from fungal infection through competition with the pathogen for nutrients, the production of antifungal metabolites, parasitism (lysis of the pathogen), or through enhancement of the plant resistance mechanisms [11,27]. The growth of *P. aphanidermatum* was successfully inhibited by *P. oligandrum* isolates with growth reductions reached 71.3% with *P. oligandrum* MS15. The millipore sterilized half-strength filtrate of the bioactive *P. oligandrum* MS15 showed more activity (34.1%) compared with autoclaved filtrates in the linear growth reduction of pathogenic *P. aphanidermatum*, which reach 29.4%. These results were confirmed through the reduction of the mycelial dry weight of pathogenic *P. aphanidermatum* with half-strength millipore sterilized filtrates of the bioactive *P. oligandrum* MS15 to 40.11% compared with autoclaved filtrate (35.7%). That explains the low activity of autoclaved *P. oligandrum* filtrates, which may affect and destroy the active metabolites [28,29,30,31].

*P. oligandrum* isolates (MS15, MS19, MS31) parasitized hyphae of *P. aphanidermatum* by entangling the hyphae of *P. aphanidermatum* with thin haustorial branches or infection pegs, eventually leading to destruction, revealing the mode of action of antagonism between *P. oligandrum* and *P. aphanidermatum* [20,29] as appears in our results of screening for hyphal interaction under light and scanning microscopy. There were at least three known modes of *P. oligandrum* action on plant pathogens: mycoparasitism mediated by intimate hyphae interactions, antibiosis and enhancement of plant resistance due to protein metabolite (oligandrin) production [8]. The coiling and penetration of *P. oligandrum* hyphae to *P. aphanidermatum* hyphae observed in this study had frequently been reported [17,20]. Additionally, the isolate MS15 of *P. oligandrum* was the most effective antagonistic one and reduced the disease severity of *P. aphanidermatum* infected *G. max* to 93.3% in agar bottles and 80% in soil pots assay. These results confirm the ability of *P. oligandrum* to significantly reduce the infection by *P. aphanidermatum* [14,21]. Moreover, the biocontrol agent *P. oligandrum* was proved to enhance the growth of *G. max* seedling. These growth promotions of *P. oligandrum* may be attributed to the elicitation of bioactive fungal metabolites [14,15]. It can be concluded that *P. oligandrum* isolated in this study worked as mycoparasite and antifungal metabolites producer, which suppresses the plant-parasitic *P. aphanidermatum*.

The present study confirmed the efficiency and importance of *P. oligandrum* as a biological control against root diseases avoiding biohazards of fungicides usages.

## 4. Materials and Methods

The method of zoospores baiting technique (ZBT) was used for isolation of the biocontrol agent *P. oligandrum* during the survey of *Pythium* species associated with the rhizosphere soil of *Raphanus sativus*, *Eruca sativa,* and *Allium cepa* in El-Minia governorate, Egypt during the spring of 2016. The isolation was done using Nystatin Ampicillin Rifampcin Miconazole (NARM) selective medium which was described by Senda et al. [32].

Rotten roots and basal parts of diseased seedling of *G. max* were collected in March of 2016 from a field in Kidwan, El-Minia governorate, Egypt (Figure 1) and used for isolation of pathogenic *P. aphanidermatum*. The deceased tissues were cleaned with 50% ethanol (*v*/*v*) for 30 s accompanied by washing with sterile distilled water, then dried with sterile filter paper and transported to NARM medium [32]. All seeded plats were incubated at 20 °C and examined daily. Obtained colonies were then purified and subjected to identification.

### 4.1. Identification of Pythium Species

NARM selective medium was found to repress the bacterial growth in *Pythium* cultures whilst not affecting *Pythium* itself. The required reproductive fungal structure including the shape, size, and position of sporangia, antheridia, oogonia, oospores, and zoospores formation was ascertained in grass blade culture [14,16]. The morphological identification of isolated *Pythium* species was carried out using the keys of Dick and Plaats-Niterink [7,33]. In addition, *Pythium* species identification was confirmed through the DNA extraction and amplification using polymerase chain reaction (PCR) technique according to [32,34], then the sequencing of rDNA-ITS regions including the 5.8 SrDNA, were analyzed according to the method developed by [21,35] using the basic local alignment search tool (BLAST).

### 4.2. Antagonistic Activity of P. oligandrum Isolates against the Pathogenic P. aphanidermatum Using Dual Culture Technique

Three isolates of *P. oligandrum* (MS15, MS19, and MS31) were identified as biocontrol agents. Sterilized PDA plates were inoculated by 5 mm discs cutting from the margins of each of the pathogenic *P. aphanidermatum* isolate and the three isolates of the biocontrol *P. oligandrum* at a distance of 4 cm from each other. Three replicates were managed per each treatment and the plates were incubated at 25 °C for 3–6 days. When the growth of control *P. aphanidermatum* mycelia covers all plates surface, the data were recorded. Percentages of reduction in mycelial growth of the pathogenic *P. aphanidermatum* were calculated and recorded in relation to the control.

### 4.3. Effect of Half-Strength Culture Filtrate of P. oligandrum on the Linear Growth of Pathogenic P. aphanidermatum

Sterilized filtrate of 5-day old cultures of *P. oligandrum* isolates grown on potato dextrose broth (PDB) was tested against the growth of pathogenic *P. aphanidermatum*. The tested filtrates were sterilized by either autoclave or using a 0.22 μm millipore filter. Equal volumes of both sterilized filtrates of *P. oligandrum* isolates and sterilized double strength PDA medium were mixed (1:1, *v*/*v*) before pouring in sterilized Petri dishes. Discs of the 3-day old culture of the pathogenic *P. aphanidermatum* were put in the middle of these plates and incubated at 25 °C. Data were recorded when the mycelial growth of the control *P. aphanidermatum* covers all surfaces of plates and of comparing with control, the reduction mycelial growth percentages were recorded [11].

### 4.4. Effect of Half-Strength Culture Filtrate of P. oligandrum on the Dry Weight of Pathogenic P. aphanidermatum

An equal volume of sterilized filtrates (ether by Autoclave or 0.22 Mm millipore) at a rate of 50 mL was mixed with 50 mL sterilized double strength of potato dextrose broth (PDB) in 250 mL Erlenmeyer flasks. Three control flasks containing 50 mL double-strength PDB medium mixed with 50 mL of sterile distilled water. Mycelial discs of 5 days old culture of the phytopathogenic *P. aphanidermatum* were inoculated and the flasks were incubated at 25 °C for 8 days. Mycelium from each flask was filtered using Whatman’s No. 2 filter paper, washed and dried in the oven at 80 °C for 72 h, and finally weighed. All the cultures were maintained in three replicates. The percent inhibition in mycelial dry weight was recorded to indicate the effect of the antagonist’s filtrate on the pathogenic fungal growth.

### 4.5. Mycoparasitism between P. oligandrum and P. aphanidermatum Using SEM

Mycelial strips were obtained from the contact area between the two colonies of *P. oligandrum* and *P. aphanidermatum* grown on CMA for three days at 25 °C and then fixed in 2.5% gluteraldehyde-sodium phosphate buffer (0.13 M, Ph 7.2) overnight. The specimens were washed three times with buffer and then postfixed in osmium tetraoxide for 2 h, washed again with buffer, and dehydrated using ascending series of ethanol level (30–100%) for 15 min at each step. The specimens were dried at the critical point and coated with carbon to be examined using the scanning electron microscope (JEOL JSM-6380 LA) operating at 20 kV [30].

### 4.6. Pathogenicity of P. aphanidermatum and Its Possible Control Measure

#### 4.6.1. In Agar Bottles Assay

Pathogenicity and possible control of *P. aphanidermatum* using *P. oligandrum* were investigated according to the method described by Abdelzaher and Kageyam [23]. *G. max* seeds were surface sterilized and germinated to form radicals and plumules for 3 days at 25 °C, then three viable seeds were grown onto 2% WA in a 250 mL Erlenmeyer flask. Three seedling flasks were inoculated by three 5-mm discs cutting from a 5-days old culture of pathogenic *P. aphanidermatum* to evaluate their pathogenicity as a positive control. Other seedling flasks were inoculated by three 5-mm discs of isolates of biocontrol agent *P. oligandrum* isolates act as a negative control. While the other seedling flasks were inoculated by three discs of each of *P. oligandrum* isolates and *P. aphanidermatum* to evaluate the bioactivity of *P. oligandrum* isolates (MS15, MS19, and MS31). The inoculated flasks were incubated in a growth cabinet at 25 °C with a 12 h photoperiod (91 µmol m-2S-1). Damping-off was restricted as the difference between seedlings emergence in non-inoculated controls and inoculated one [21]. The treated seedlings flasks were observed to detect damping-off diseases by recording seedling emergence.

#### 4.6.2. In Soil Pots Assay

To prepare *P. aphanidermatum* inoculum, five grams of grass blade leaf fragments (0.5 cm × 1 cm) and 2 gm glucose were saturated with 10 mL distilled water in a 250 mL Erlenmeyer flask. After autoclaving, each flask was inoculated with three discs of *P. aphanidermatum*, then incubated at 25 °C for 7 days. Inoculum concentration of 2.5%, was taken by mixing 1 gm of colonized grass leaf fragments in the Erlenmeyer flask with 50 gm of oven-dried (70–80 °C for 2 days) loam sandy soil (LSS) using a sterilized mortar. Two and a half gm of this mixture was added to 97.5 gm of sterilized soil (LSS). Inoculated soil was placed in plastic pots and exterior sterilized seeds of *G. max* were sowed after viability test as three seeds in each pot. The pots were acted as a positive control of pathogenicity and incubated in a growth cabinet. Damping-off disease severity was determined as mentioned above.

To evaluate the efficacy of *P. oligandrum* against the damping-off disease of *G*. *max* in artificially infested soil with *P. aphanidermatum*, mycelial mats of *P. oligandrum* isolates were got from cultures developed in V-8 juice broth incubated for 10 days in darkness at 25 °C, then the mycelial mats were cleaned with sterile saline water and fragmented by tissue homogenizer. The preparation was utilized to coat *G. max* seeds using 3% carboxy methyl cellulose solution (CMC). Mycelial mats of *P. oligandrum* isolates (MS15, MS19, and MS31) at concentrations of 1000 prop gules per ml were mixed with exterior sterilized seeds of G. max. The seeds were spread in the sterile opened Petri dish to dry overnight in a refrigerator at 5 °C. Three coated seeds of *G. max* were sown into *P. aphanidermatum* infested soil at 5 mm depth from the soil surface as treated pots.

Some non-coated *G. max* seeds have been sown into infested soil with *P. aphanidermatum* and used as a positive control. Another group of coated seeds with the biocontrol *P. oligandrum* was sown in sterilized soil as a negative control. All pots were put in a growth cabinet as reported previously. Numbers of *G. max* seeds, which damped-off were counted after 14 days from sowing. Experiments were done with ten replicate pots per treatment.

### 4.7. Effects of G. max Seeds Treatment with P. aphanidermatum and Three Isolates of Bioagent P. oligandrum on Growth Seed Parameters

The growth promotion percentage for the shoot length, root length, and the number of lateral roots as growth parameters of the treated plants with the phytopathogenic *P. aphanidermatum* and *P. oligandrum* isolates were measured and calculated according to the formula:

(1) $$The\;growth\;promotion\;percentage=\frac{Plant\;growth\;of\;treatment\;-\;Plant\;growth\;of\;control}{Plant\;growth\;of\;control}\;\times \;100$$

Statistical analyses software, GraphPad Prism 5.0 (GraphPad Software, Inc., La Jolla, CA, USA), was used to statistically analyze the data of antagonistic activity using one-way analysis of variance and Tukey’s test. The mean of triplicates was used to tabulate the data (*p* < 0.05).

## 5. Conclusions

The results attained by the present study conclude that *P. oligandrum* exhibited highly potential bioactivity against *P. aphanidermatum* mainly through mycoparasitism. Bioassay of *P. oligandrum* as a control agent showed that bioactivity of *P. oligandrum* using the dual culture technique was more effective than that of fungal filtrate. The confirmed efficiency of *P. oligandrum* as a biological control against *G. max* root diseases avoid the biohazards of fungicides application. This efficiency of *P. oligandrum* as a biological control agent increases the ability of its usage as an effective natural alternative to chemical fungicides used to control *G. max* damping-off disease.

## Figures and Tables

**Table 1:** The reduction in mycelial growth of the pathogenic *P. aphanidermatum* by *P. oligandrum* isolates (MS15, MS19_,_ and MS31).

Incubation Period	Antagonistic isolates	*P. aphanidermatum*	
		Linear Growth (mm)	Reduction (%)
	*P. oligandrum* MS15	29.52 * ± 0.13	67.2
3 days	*P. oligandrum* MS19	30.51 * ± 0.16	66.1
	*P. oligandrum* MS31	31.23 * ± 0.15	65.3
	*P. oligandrum* MS15	25.83 * ± 0.14	71.3
6 days	*P. oligandrum* MS19	27.01 * ± 0.05	67.1
	*P. oligandrum* MS31	28.17 * ± 0.08	68.7
Control of *P. aphanidermatum*		90.00 * ± 0.02	----

Data are means of (3) replicates ± the standard error of the three isolates; values followed by an asterisk (*) are significantly compared with control at *p* ≤ 0.05.

**Table 2:** Effect of half-strength culture filtrates of *P. oligandrum* isolates (MS15, MS19 and MS31) on mycelial linear growth of the pathogenic *P. aphanidermatum*.

Type of Culture Filtrate Sterilization	Antagonistic Isolates	*P. aphanidermatum*	
		Linear Growth (mm)	Reduction (%)
Millipored filtrate	*P. oligandrum* MS15	59.24 * ± 0.15	34.18
	*P. oligandrum* MS19	60.73 * ± 0.08	32.52
	*P. oligandrum* MS31	61.46 * ± 0.14	31.71
Autoclaved filtrate	*P. oligandrum* MS15	63.48 * ± 0.17	29.47
	*P. oligandrum* MS19	65.58 * ± 0.09	27.13
	*P. oligandrum* MS31	65.84 * ± 0.11	26.84
Control of *P. aphanidermatum*		90.00 * ± 0.02	–

Data are means of (3) replicates ± the standard error of the three isolates; values followed by an asterisk (*) are significantly compared with control at *p* ≤ 0.05.

**Table 3:** Effect of half-strength culture filtrate of *P. oligandrum isolates* (MS15, MS19 and MS31) on the mycelial dry weight of the pathogenic *P. aphanidermatum*.

Type of Filtrate Sterilization	Antagonistic Isolates	*P. aphanidermatum*	
		Mycelial Dry Weight (mg)	Inhibition (%)
Millipored filtrate	*P. oligandrum* MS15	463.62 * ± 0.17	40.11
	*P. oligandrum* MS19	484.53 * ± 0.12	37.4
	*P. oligandrum* MS31	489.19 * ± 0.15	36.8
Autoclaved filtrate	*P. oligandrum* MS15	497.68 * ± 0.18	35.7
	*P. oligandrum* MS19	529.42 * ± 0.19	31.6
	*P. oligandrum* MS31	542.57 * ± 0.11	29.9
Control *P. aphanidermatum*		774.00 *± 0.14	–

Data are means of (3) replicates ± the standard error of the three isolates; values followed by an asterisk (*) are significantly compared with control at *p* ≤ 0.05.

**Table 4:** Effect of *P. oligandrum* isolates on *G. max* pre-emergence damping-off disease grown in 2% water agar and in soil pots.

Treatments	*Glycine max* Seedlings			
	2% Water Agar		Soil Pots	
	Inhibition (%)	Survival (%)	Inhibition (%)	Survival (%)
Control (No *Pythium*)	0	100	00	100
*P. aphanidermatum*	100	00	86.7	13.3
*P. oligandrum* MS15	0	100	0	100
*P. oligandrum* MS19	0	100	0	100
*P. oligandrum* MS31	0	100	0	100
*P. aphanidermatum* + *P. oligandrum* MS15	0.67	93.3	20	80
*P. aphanidermatum* + *P. oligandrum* MS19	12.7	87.2	24.9	75.1
*P. aphanidermatum* + *P. oligandrum* MS31	13.3	86.7	23.3	76.7

**Table 5:** Effects of *G. max* seed treatment with three isolates of bioagent *P. oligandrum* on the growth seed parameters.

Seed Treatments	Plant Growth Parameters			Percentage of Plant Growth Promotion		
	Shoot Length (cm)	Root Length (cm)	No. of Lateral Roots	Shoot Length (%)	Root Length (%)	No. of Lateral Roots (%)
Control (No *Pythium*)	17.1 * ± 0.7	4.3 * ± 0.6	11.7 * ± 1.1	00	00	00
*P. oligandrum* MS15	18.7 * ± 1.1	8.5 * ± 0.9	22.3 * ± 1.3	8.6	97.7	90.6
*P. oligandrum* MS19	17.9 * ± 0.9	7.1 * ± 1.3	19.4 * ± 0.9	4.7	65.1	65.8
*P. oligandrum* MS31	18.5 * ± 1.2	7.8 * ± 0.8	21.5 * ± 1.2	8.2	81.4	83.8

Data are means of (3) replicates ± the standard error of the three isolates; values followed by an asterisk (*) are significantly compared with control at *p* ≤ 0.05.
